# Zero Valent Iron Significantly Enhances Methane Production from Waste Activated Sludge by Improving Biochemical Methane Potential Rather Than Hydrolysis Rate

**DOI:** 10.1038/srep08263

**Published:** 2015-02-05

**Authors:** Yiwen Liu, Qilin Wang, Yaobin Zhang, Bing-Jie Ni

**Affiliations:** 1 Advanced Water Management Centre, The University of Queensland, St Lucia, QLD 4072, Australia; 2 Key Laboratory of Industrial Ecology and Environmental Engineering, Ministry of Education, School of Environmental Science and Technology, Dalian University of Technology, Dalian 116024, China

## Abstract

Anaerobic digestion has been widely applied for waste activated sludge (WAS) treatment. However, methane production from anaerobic digestion of WAS is usually limited by the slow hydrolysis rate and/or poor biochemical methane potential of WAS. This work systematically studied the effects of three different types of zero valent iron (i.e., iron powder, clean scrap and rusty scrap) on methane production from WAS in anaerobic digestion, by using both experimental and mathematical approaches. The results demonstrated that both the clean and the rusty iron scrap were more effective than the iron powder for improving methane production from WAS. Model-based analysis showed that ZVI addition significantly enhanced methane production from WAS through improving the biochemical methane potential of WAS rather than its hydrolysis rate. Economic analysis indicated that the ZVI-based technology for enhancing methane production from WAS is economically attractive, particularly considering that iron scrap can be freely acquired from industrial waste. Based on these results, the ZVI-based anaerobic digestion process of this work could be easily integrated with the conventional chemical phosphorus removal process in wastewater treatment plant to form a cost-effective and environment-friendly approach, enabling maximum resource recovery/reuse while achieving enhanced methane production in wastewater treatment system.

Large amounts of organic matter from wastewater are converted into waste activated sludge (WAS) during biological treatment processes in wastewater treatment plants (WWTPs)^1^. Anaerobic digestion of WAS has been widely applied to stabilize and reduce the volume of WAS as well as produce a renewable bioenergy resource in the form of methane^2^,^3^,^4^,^5^. The anaerobic digestion process generally consists four stages, i.e. hydrolysis, fermentation, acetogenesis and methanogenesis for methane production^6^. However, the application of anaerobic digestion is often limited by the slow hydrolysis rate and/or poor biochemical methane potential (or degradation extent) of the WAS^7^,^8^,^9^,^10^,^11^.

In order to effectively enhance methane production from WAS in anaerobic digestion, a number of strategies have been developed, such as thermal, chemical, and mechanical methods^12^,^13^,^14^,^15^,^16^,^17^,^18^,^19^,^20^,^21^. However, most of these methods are cost intensive owing to high energy input or large chemical requirements^7^,^22^,^23^. Thus, alternative cost-effective approach to improve methane production from WAS in anaerobic digestion process is highly desired.

Zero valent iron (ZVI), a cheap reducing agent, has been widely used in wastewater pretreatment, groundwater purification and soil remediation^24^,^25^. Recent studies have found that ZVI addition in anaerobic reactors for biological wastewater treatment could significantly improve chemical oxygen demand (COD) removal by ca. 25%^26^,^27^,^28^,^29^. Indeed ZVI can lower oxidation–reduction potential and serve as an acid buffer, thus helping maintain a stable and favourable condition for methanogens. Previous studies also demonstrated that ZVI addition could promote hydrolysis/acidification and optimize volatile fatty acid (VFA) compositions^30^,^31^. Therefore, ZVI addition to the anaerobic digester could be a potential cost-effective approach to improve methane production from WAS^32^,^33^.

In this work, the impacts of three different types of ZVI (i.e., iron powder, clean scrap and rusty scrap) on methane production from WAS in anaerobic digestion were evaluated systematically using both experimental and mathematical approaches. A model-based analysis was performed to reveal the mechanism of ZVI-driven enhancement of methane production from WAS. Based on the results of economic analysis, a cost-effective integrated ZVI-based anaerobic WAS digestion process was also proposed.

## Results

### Effects of ZVI addition on methane production

**Figure 1:**
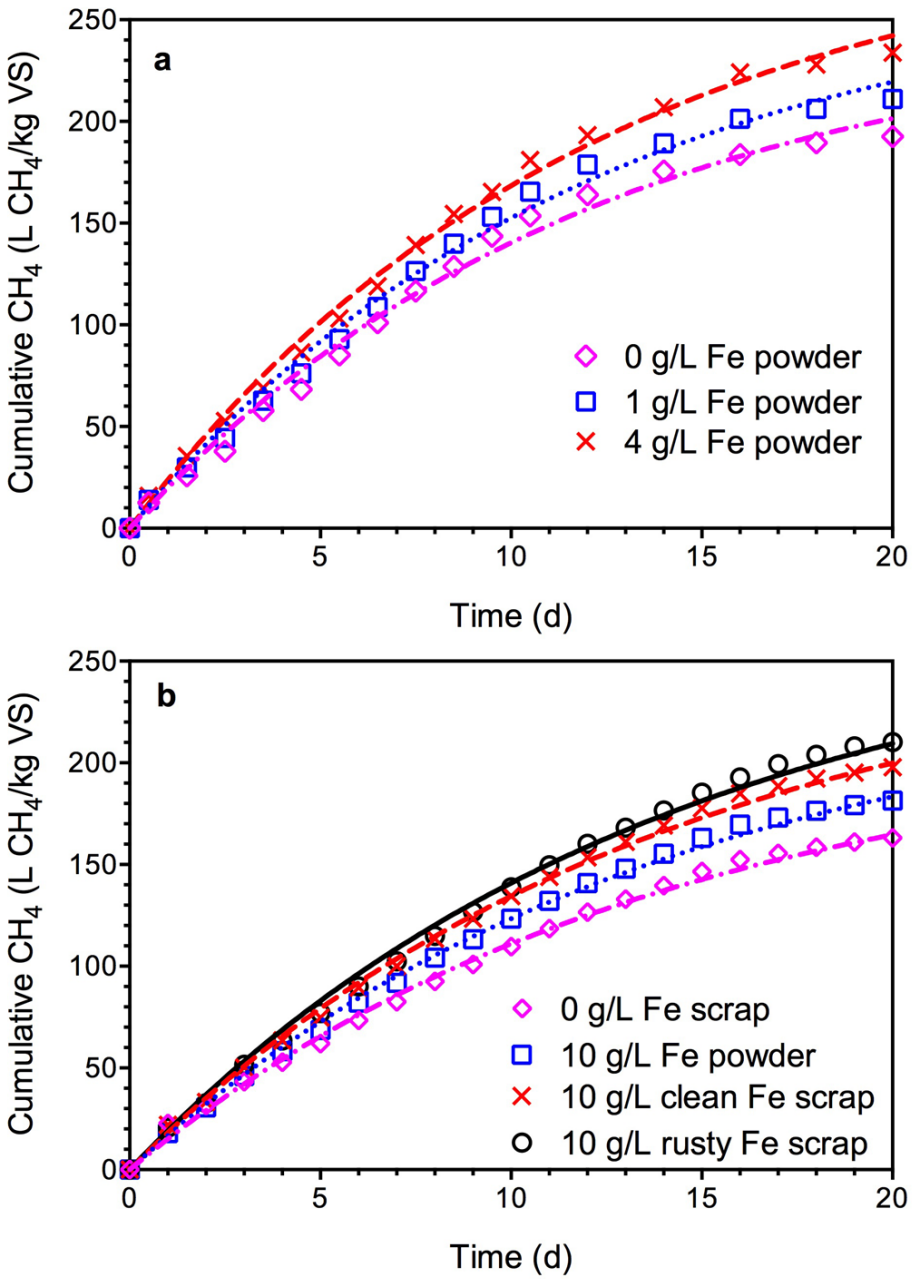
The measured and simulated methane production in the BMP tests (symbols represent experimental measurements and lines represent model simulations): (a) data from Experiment I; and (b) data from Experiment II.

Three types of ZVI were evaluated, i.e., iron powder, clean scrap and rusty scrap. Fig. 1 presents the methane production results from the biochemical methane potential (BMP) tests in Experiment I and II (see Methods Section). In general, ZVI addition enhanced the methane production from WAS. The increased ZVI powder addition resulted in increasing methane production (Fig. 1a). For example, 4 g/L ZVI addition increased methane production by 21% as compared to the control (0 g/L ZVI powder addition) on Day 20. As shown in Fig. 1b, 10 g/L ZVI powder, 10 g/L clean iron scrap and 10 g/L rusty iron scrap led to 11%, 22% and 30% increase on methane production, respectively, compared to that of control on Day 20. The level of methane production variation between Experimental I and II is not unexpected as real sludge from a full-scale WWTP was used, with characteristics likely varying with time. Therefore, direct comparisons between Experiment I and II are not meaningful due to the possible variation of the sludge characteristics during sludge sampling. In addition, it should be noted that the methane production by utilization of ZVI as electron donors is negligible compared to the overall methane production in the system as ZVI powder or clean scrap addition produces similar content of methane with the addition of rusty scrap.

These results demonstrated that ZVI addition indeed enhanced methane production from WAS during anaerobic digestion. The results also showed that both the clean and the rusty iron scrap were more effective than the iron powder for improving methane production from WAS. The better performance of ZVI scrap was likely due to its better contact with sludge and liquid^33^. In particular, the addition of rusty iron scrap is the most effective ZVI form for improving methane production from WAS, likely due to the fact that Fe (III) oxides on the rusty iron scrap surface could induce dissimilatory ferric iron reduction to enhance degradation of complex substrates such as WAS^34^.

### Effects of ZVI on hydrolysis rate and biochemical methane potential

The hydrolysis rate (k) and biochemical methane potential (B_0_) were estimated using both one-substrate and two-substrate models. Table 1 shows the estimated k and B_0_ for the methane production from the WAS digestion subject to different ZVI forms and dosages using one-substrate model, while the estimated values of k,_rapid_, k,_slow_, B_0,rapid_ and B_0,slow_ in both Experiment I and II using two-substrate model are presented in Table S1 in Supplementary Material. Overall, k,_rapid_ and B_0,rapid_ in two-substrate model are the same as k and B_0_ in one-substrate model. Both k,_slow_ and B_0,slow_ in two-substrate model are zero after fitting. These modeling results indicated that the WAS composition was homogeneous and the methane production from the WAS could be well described by one-substrate model.

The simulated methane production curves using one-substrate model are shown in Fig. 1, which matched all the experimental data from both Experiment I and II, further confirming the one-substrate model could well describe the methane production data. As can be seen from Table 1, the ZVI addition at all the levels applied achieved significantly higher B_0_ than that of the control. The biochemical methane potential was enhanced by 9%–21% in Experiment I and 12%–29% in Experiment II compared to the corresponding control. In contrast, the ZVI addition has no effect on the k value and the obtained k values were constant in both Experiment I (ca. 0.083 d^−1^) and Experiment II (ca. 0.072 d^−1^) regardless of the amount of ZVI addition.

**Figure 2:**
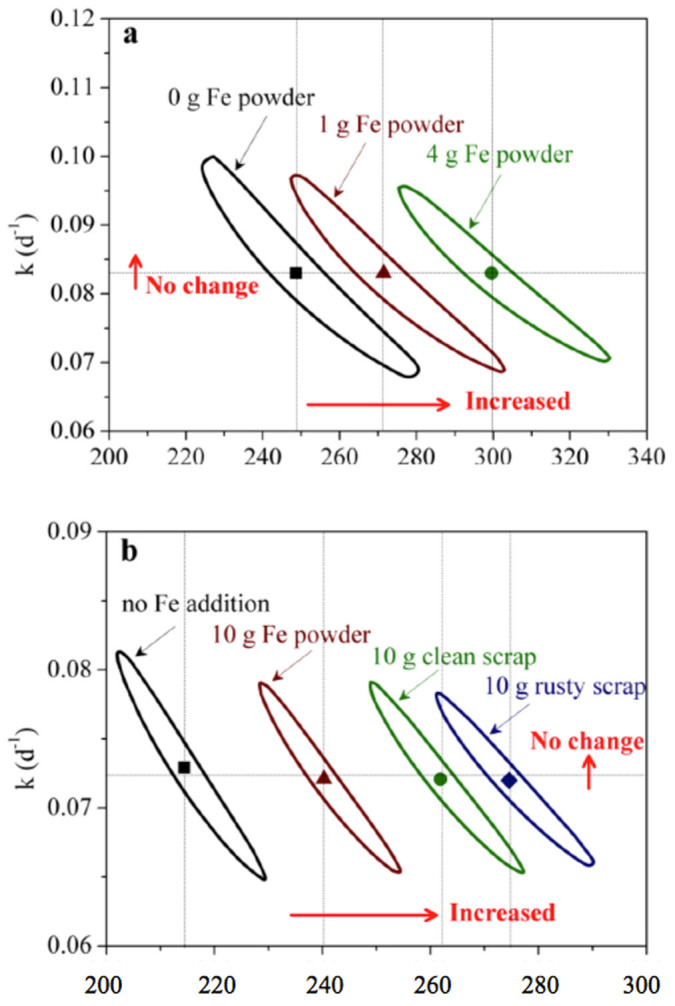
The 95% confidence regions of the estimated hydrolysis rate (k) and biochemical methane potential (B_0_) with different ZVI additions: (a) using data from Experimental I; and (b) using data from Experiment II.

Fig. 2 shows the 95% confidence regions of k and B_0_, which provide valuable information about model uncertainty and the identifiability of the obtained parameter values. The increased ZVI addition consistently resulted in better biochemical methane potential (B_0_), and the confidence region moved rightward to the higher B_0_ direction (x-axis) in Fig. 2. In contrast, the increased ZVI addition had no impact on the hydrolysis rate, with no real changes in confidence region locations on y-axis. In addition, there was no obvious increase in confidence region area in both Fig. 2a and 2b.

## Discussion

### ZVI addition improved biochemical methane potential of WAS rather than its hydrolysis rate

There are two key measures of sludge degradability that are relevant, the apparent first order degradation rate coefficient (k) and the biochemical methane potential (B_0_), which represent the speed and extent of sludge conversion, respectively^35^. Model-based analysis of these two parameters and the related parameter identifiability in this work clearly showed that ZVI addition significantly enhanced methane production from WAS through improving the biochemical methane potential of WAS rather than its hydrolysis rate.

**Figure 3:**
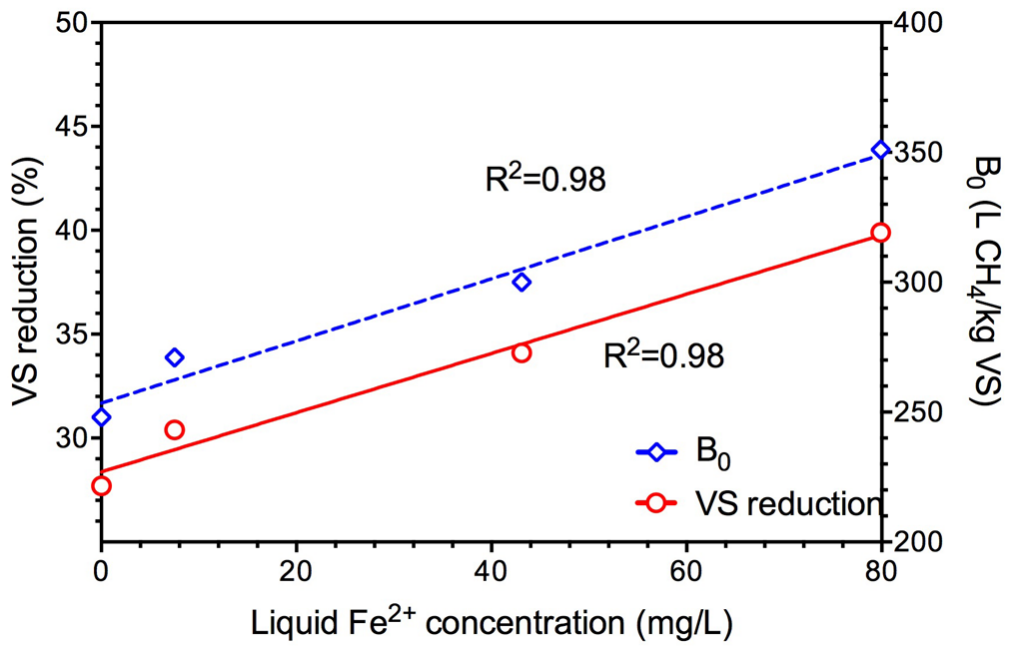
Relationships between the released ferrous iron concentrations and the percentage of VS reduction as well as the obtained B_0_ value in Experiment I.

Feng et al.^32^ did not look into the mechanisms for the enhanced methane production by ZVI addition and only hypothesized that the main reason might be the improved major enzyme activities related to hydrolysis and acidification. Contradictorily, this study demonstrated that the ZVI addition did not accelerate the hydrolysis rate (k) in both experiments with different types of ZVI addition. On the contrary, biochemical methane potential (B_0_) was significantly improved by ZVI addition, indicating that ZVI increased the extent of sludge conversion and altered the sludge property^35^. It has been reported that VS destruction during anaerobic digestion of waste activated sludge generally increased with the increase of ferrous iron content in the sludge^36^,^37^. Indeed, ZVI can release from Fe^0^ to Fe^2+^ (Fe^0^ + 2H^+^ = Fe^2+^ + H_2_), and thus leading to a significant increase of iron content in the sludge^33^,^38^. As shown in Fig. 3, in this work, the released ferrous iron concentrations from ZVI also showed a good correlation with both VS reduction and the biochemical methane potential (B_0_). Therefore, the alternation of sludge property to improve biochemical methane potential by ZVI could likely be the main reason for the enhanced performance of methane production.

### A strategy to implement ZVI-based anaerobic digestion process in wastewater treatment plant

From an integrated environmental and economic perspective, nutrients source in wastewater treatment systems should be managed such that both good nutrients removal performance and high resource recovery or reuse can be achieved. Based on the findings of this work, a new strategy could be proposed to simultaneously enhance methane production from WAS and iron resource reuse through integrating the ZVI-based anaerobic digestion process of this work with the conventional chemical phosphorus removal process in WWTPs.

**Figure 4:**
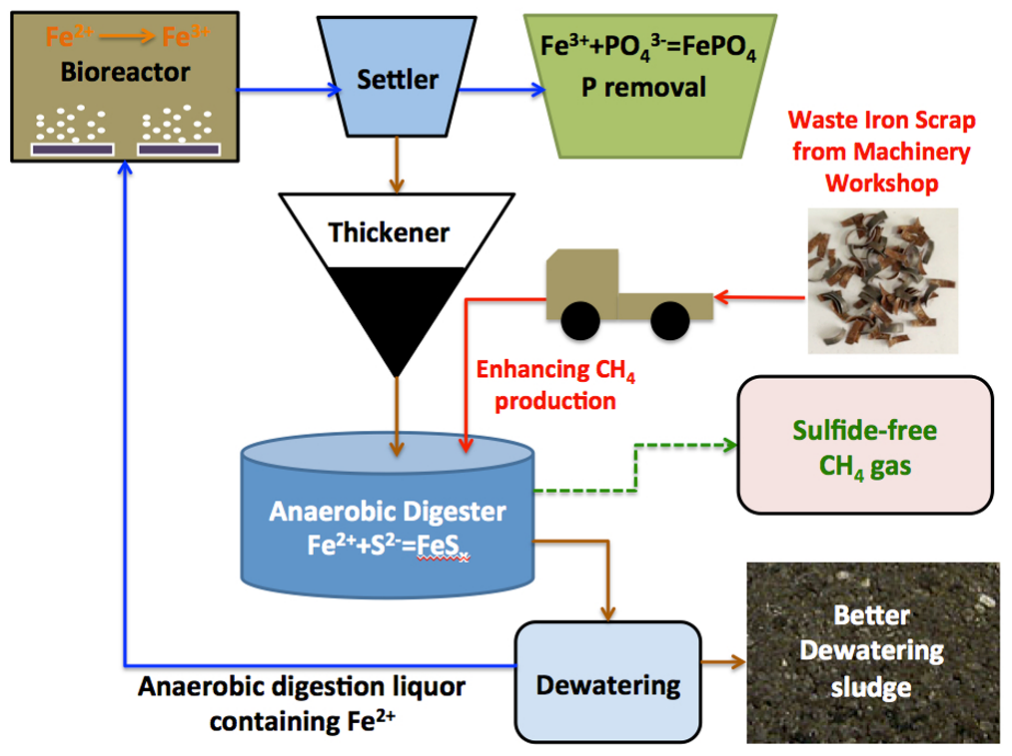
A proposed strategy to integrate ZVI-based anaerobic digestion process of this work with the conventional chemical phosphorus removal process in wastewater treatment plant. ZVI addition in anaerobic digester can enhance methane production from WAS. The sulfide produced in anaerobic digester can be precipitated by ferrous iron that produced from ZVI addition, resulting in enhanced sulfide-free biogas (methane) production. The anaerobic digestion liquor containing Fe (II) can be reused and fed into bioreactors, in which the Fe (II) can be oxidized to Fe (III). The generated Fe (III)-containing effluent can then be used for chemical phosphorus removal process, to form a cost-effective and environment-friendly technology, enabling maximum resource recovery/reuse while achieving enhanced methane production in wastewater treatment system.

As presented in Fig. 4, waste iron scrap (the most efficient ZVI as demonstrated in this work) can be freely obtained from machinery factory and then transported to the WWTP. The obtained iron scrap (ZVI) can be added to the anaerobic digester in order to enhance the methane production by increasing the biochemical methane potential. In anaerobic digester, ZVI can be released from Fe^0^ to Fe^2+^, and thus eliminated the potential sulfide production/accumulation issues as well as the possible H_2_S emission in the biogas in traditional anaerobic digester through iron sulfide precipitation^39^. This in turn could further enhance the performance of WAS digestion without additional chemical cost from external ferrous/ferric iron dosing^40^. With regard to the generation of organic sulfur odors from the dewatered sludge cakes, iron could also reduce odor-causing gases, resulting in better quality of dewatering sludge. More importantly, the Fe (II) in anaerobic digestion liquor can be recycled to bioreactor and further oxidized to Fe (III), which can be used for chemical phosphors removal via the generation of FePO_4_^41^. This strategy would not only represent a significant process cost reduction (further discussed below), but also improve the sludge and wastewater treatment efficiency, enabling maximum resource (iron) reuse while achieving improved methane production. In addition, from a network-wide view, commonly used ferric iron dosing in sewers for H_2_S control^42^ might also be useful for CH_4_ production enhancement during anaerobic digestion and phosphors removal in the WWTP.

### Potential economic feasibility of ZVI-based technology for enhancing biological methane production

It has been demonstrated that the estimated lab-scale BMP results are more conservative or comparable to full-scale test results^43^. Thus, the estimated values obtained in the current study are used for a conservative assessment of the potential economic feasibility of the proposed ZVI-based anaerobic digestion technology. This was carried out by a desktop scaling-up study on a full-scale WWTP with a population equivalent (PE) of 400,000 and with an anaerobic sludge digester at a hydraulic retention time (HRT) of 20 days. 10 g/L rusty iron scrap was chosen for the following economic evaluations.

From the Fe^2+^ released (41 mg/L), theoretically, the iron scrap could be recycled for approximately 243 batches (10*1000/41) if the loss of iron solid through effluent is ignored. With a 29% increase in methane production at this level of ZVI addition, the net economic benefit is estimated to be around $231,000 per annum compared with the system without ZVI addition (see Table S2 in Supplementary Material). The net benefit arises from the enhanced methane production associated benefit (i.e., its conversion to heat and power) ($150,000 per annum) and decreased WAS transport and disposal costs ($90,000 per annum) overweighing the additional costs for ZVI transport and ZVI chamber ($9,000 per annum). The advantages of ZVI addition on sulfide control in digester, phosphors removal through anaerobic digestion liquor recycle and better dewatering sludge have not been considered. Therefore, the ZVI-based technology is potentially economically attractive indeed. However, the benefit and cost values presented should be considered indicative only. In particular, they may vary from region to region and from country to country, depending on the local conditions. In addition, the direct quantitative economic and performance comparison with other available technologies are difficult at this stage since the results depend on many factors including the WAS characteristics among others^23^, which remains further investigations in the future. This could and should be done in future studies by performing experiments using the same WAS and under similar operating conditions.

Moreover, it should be noted that there is no environmental consequence of the proposed chemical-free ZVI-based technology based on CO_2_ emission, revealing this approach being environmental friendly. In comparison, some other WAS pretreatment technologies (i.e., thermal and alkaline pretreatment) might cause negative environmental effect^44^. Different from temperature phased anaerobic digestion and mechanical pretreatment which generally increase k^45^,^46^, this ZVI-based approach improved B_0_, thus potentially allowing more methane production in terms of performance improvements for anaerobic digesters. Since it does not improve the degradation rate, this requires the same amount of HRT in order to achieve maximized sludge reduction. It should be noted that lab-scale batch tests were performed in our study. Full-scale system may behave differently in terms of k and B_0_ as demonstrated in Bastone et al.^43^. Therefore, full-scale trials are needed to further evaluate this technology.

In summary, the effects of three different types of ZVI (i.e., iron powder, clean scrap and rusty scrap) on methane production from WAS in anaerobic digestion were investigated by using both experimental and mathematical approaches. The results demonstrated that both the clean and the rusty iron scrap were more effective than the iron powder for improving methane production from WAS. ZVI addition significantly enhanced methane production from WAS through improving the biochemical methane potential of WAS rather than its hydrolysis rate. The alternation of sludge property by ZVI resulted in improved biochemical methane potential and thus the enhanced methane production. The ZVI-based anaerobic digestion process could be potentially implemented and integrated with the conventional chemical phosphorus removal process in wastewater treatment plant to form a cost-effective and environment-friendly technology, enabling maximum resource recovery/reuse while achieving enhanced methane production in wastewater treatment system.

## Methods

### Waste activated sludge

The waste activated sludge used in this work was collected from the sludge treatment unit at a full-scale municipal wastewater treatment plant in Dalian, China. The sludge was stored at 4°C before use. The volatile solids (VS) to total chemical oxygen demand (TCOD) ratios of the sludge used for methane production ranged between 0.60 and 0.67.

### ZVI sources

Three types of ZVI were evaluated, i.e., iron powder, clean scrap and rusty scrap. The ZVI powder has a diameter of 0.2 mm with BET surface area of 0.05 m^2^/g and purity >98%. The rusty scrap (about 8 mm * 4 mm * 0.5 mm, purity > 95%) was obtained from a machinery workshop in Dalian, China. The clean scrap was acquired through a pretreatment of the rusty scrap to remove the rusty cover. The difference between the two scraps is that the rusty scrap had a corrosion layer covering the surface of the scrap^33^.

### Anaerobic biochemical methane potential tests

In order to evaluate the effect of different forms of ZVI on methane production in anaerobic digestion, methane production from the WAS with different types of ZVI addition was assessed using anaerobic batch BMP tests^32^. The inoculum for the BMP tests was collected from an anaerobic digester^33^. Two types of batch experiments were performed. In Experiment I, 0, 1.0, and 4.0 g/L of ZVI powder were added into three identical sets of BMP test vials, respectively. In Experiment II, 10 g/L ZVI powder, 10 g/L clean scrap and 10 g/L rusty scrap were used as ZVI sources and dosed to three identical sets of BMP vials for comparison, with a control test in which no ZVI was added.

In each test, WAS, ZVI and the inoculum obtained from the anaerobic digester were added into serum vials for BMP tests. After that, the vials were capped with silica gel stoppers. The oxygen was removed from the headspace by exchanging it with nitrogen gas for at least 10 min. All BMP tests were conducted at 35 ± 1°C for 20 d. The biogas (methane) production in BMP vials was collected and monitored by using gas chromatograph (Shimadzu, GC-14C) equipped with a thermal conductivity detector. More details of the BMP tests can be found elsewhere^32^,^33^.

### Model-based analysis

The hydrolysis rate (k) and biochemical methane potential (B_0_) are the two key parameters associated with methane production from WAS^7^,^8^,^10^. In this work, these two parameters were used to evaluate and compare the methane production kinetics and potential of the WAS at different ZVI levels or with different types of ZVI. They were estimated by fitting the methane production data from the BMP tests to a first-order kinetic model using a modified version of Aquasim 2.1d with sum of squared errors (J_opt_) as an objective function^43^. The uncertainty surfaces of k and B_0_, based on a model-validity statistical F-test with 95% confidence limits, were also estimated by using Aquasim 2.1d^43^.

Two models were applied. The first one considered a single substrate type (i.e., one-substrate model) in the first-order kinetic model^22^,^43^, as shown in Equation (1):
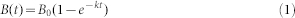
where B(t) (L CH_4_/kg VS) is the cumulative methane production at time t (d).

In the second model, the WAS samples comprised a rapidly biodegradable substrate type and a slowly biodegradable substrate type (i.e. two-substrate model)^47^. The equation of the two-substrate model is shown below:
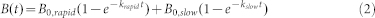
where B_0,rapid_ and B_0,slow_ (L CH_4_/kg VS) are biochemical methane potentials of the rapidly biodegradable substrates and slowly biodegradable substrates, respectively; k_rapid_ and k_slow_ (d^−1^) are hydrolysis rates of the rapidly biodegradable substrates and slowly biodegradable substrates, respectively.

Based on the determined B_0_, the degradation extent (Y) of WAS could then be calculated using Equation (3):
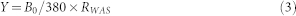
where 380 (L CH_4_/kg TCOD) is theoretical biochemical methane potential of WAS under standard conditions (25°C, 1 atm); R_WAS_ is the measured VS to TCOD ratio in the WAS.

## Author Contributions

Y.L., Q.W., Y.Z. and B.-J.N. wrote the manuscript; Y.L., Y.Z. and B.-J.N. developed the methodology; Y.L. and Q.W. performed data analysis and prepared all figures; All authors reviewed the manuscript.

## Supplementary Material

Supplementary Information SI for Zero Valent Iron Significantly Enhances Methane Production from Waste Activated Sludge by Improving Biochemical Methane Potential Rather Than Hydrolysis Rate

## Figures and Tables

**Table 1:** The estimated k and B_0_ as well as the calculated Y from Experiment I and II using one-substrate model (with 95% confidence intervals)

	k (d^−1^)	B_0_ (L CH_4_/kg VS)	Y
**Experiment I**			
0 g/L Fe powder	0.083 ± 0.007	248 ± 12	0.44 ± 0.02
1 g/L Fe powder	0.083 ± 0.006	271 ± 12	0.48 ± 0.02
4 g/L Fe powder	0.083 ± 0.005	300 ± 11	0.53 ± 0.02
**Experiment II**			
0 g/L Fe scrap	0.073 ± 0.003	214 ± 6	0.34 ± 0.01
10 g/L Fe powder	0072 ± 0.003	240 ± 5	0.37 ± 0.01
10 g/L clean Fe scrap	0.072 ± 0.003	262 ± 6	0.41 ± 0.01
10 g/L rusty Fe scrap	0.071 ± 0.003	275 ± 6	0.44 ± 0.01

## References

[b1] Duan N., Dong B., Wu B. & Dai X. High-solid anaerobic digestion of sewage sludge under mesophilic conditions: Feasibility study. Bioresour. Technol. 104, 150–156 (2012). 22104097 10.1016/j.biortech.2011.10.090

[b2] Eskicioglu C., Prorot A., Marin J., Droste R. L. & Kennedy K. J. Synergetic pretreatment of sewage sludge by microwave irradiation in presence of H_2_O_2_ for enhanced anaerobic digestion. Water Res. 42, 4674–4682 (2008). 18783812 10.1016/j.watres.2008.08.010

[b3] Tiehm A., Nickel K., Zellhorn M. & Neis U. Ultrasonic waste activated sludge disintegration for improving anaerobic stabilization. Water Res. 35, 2003–2009 (2001). 11337847 10.1016/s0043-1354(00)00468-1

[b4] Wang L., Aziz T. N. & de los Reyes F. L. Determining the limits of anaerobic co-digestion of thickened waste activated sludge with grease interceptor waste. Water Res. 47, 3835–3844 (2013). 23648286 10.1016/j.watres.2013.04.003

[b5] McCarty P. L., Bae J. & Kim J. Domestic wastewater treatment as a net energy producer–can this be achieved? Environ. Sci. Technol. 45, 7100–7106 (2011). 21749111 10.1021/es2014264

[b6] Lv W., Schanbacher F. L. & Yu Z. Putting microbes to work in sequence: Recent advances in temperature-phased anaerobic digestion processes. Bioresour. Technol. 101, 9409–9414 (2010). 20709534 10.1016/j.biortech.2010.07.100

[b7] Wang Q. *et al.* Free nitrous acid (FNA)-based pretreatment enhances methane production from waste activated sludge. Environ. Sci. Technol. 47, 11897–11904 (2013). 24041014 10.1021/es402933b

[b8] Labatut R. A., Angenent L. T. & Scott N. R. Biochemical methane potential and biodegradability of complex organic substrates. Bioresour. Technol. 102, 2255–2264 (2011). 21050752 10.1016/j.biortech.2010.10.035

[b9] Hosseini Koupaie E., Barrantes Leiva M., Eskicioglu C. & Dutil C. Mesophilic batch anaerobic co-digestion of fruit-juice industrial waste and municipal waste sludge: Process and cost-benefit analysis. Bioresour. Technol. 152, 66–73 (2014). 24280084 10.1016/j.biortech.2013.10.072

[b10] Lissens G., Thomsen A. B., De Baere L., Verstraete W. & Ahring B. K. Thermal wet oxidation improves anaerobic biodegradability of raw and digested biowaste. Environ. Sci. Technol. 38, 3418–3424 (2004). 15260343 10.1021/es035092h

[b11] Mu Y., Yu H.-Q. & Wang G. A kinetic approach to anaerobic hydrogen-producing process. Water Res. 41, 1152–1160 (2007). 17267006 10.1016/j.watres.2006.11.047

[b12] Chu L., Yan S., Xing X.-H., Sun X. & Jurcik B. Progress and perspectives of sludge ozonation as a powerful pretreatment method for minimization of excess sludge production. Water Res. 43, 1811–1822 (2009). 19282018 10.1016/j.watres.2009.02.012

[b13] Imbierowicz M. & Chacuk A. Kinetic model of excess activated sludge thermohydrolysis. Water Res. 46, 5747–5755 (2012). 22951329 10.1016/j.watres.2012.07.051

[b14] Ibeid S., Elektorowicz M. & Oleszkiewicz J. A. Modification of activated sludge properties caused by application of continuous and intermittent current. Water Res. 47, 903–910 (2013). 23219041 10.1016/j.watres.2012.11.020

[b15] Vlyssides A. G. & Karlis P. K. Thermal-alkaline solubilization of waste activated sludge as a pre-treatment stage for anaerobic digestion. Bioresour. Technol. 91, 201–206 (2004). 14592751 10.1016/s0960-8524(03)00176-7

[b16] Nah I. W., Kang Y. W., Hwang K.-Y. & Song W.-K. Mechanical pretreatment of waste activated sludge for anaerobic digestion process. Water Res. 34, 2362–2368 (2000).

[b17] Zhang D., Chen Y., Zhao Y. & Zhu X. New sludge pretreatment method to improve methane production in waste activated sludge digestion. Environ. Sci. Technol. 44, 4802–4808 (2010). 20496937 10.1021/es1000209

[b18] Zhang D., Chen Y., Zhao Y. & Ye Z. A new process for efficiently producing methane from waste activated sludge: alkaline pretreatment of sludge followed by treatment of fermentation liquid in an EGSB reactor. Environ. Sci. Technol. 45, 803–808 (2010). 21128635 10.1021/es102696d

[b19] Mehdizadeh S. N., Eskicioglu C., Bobowski J. & Johnson T. Conductive heating and microwave hydrolysis under identical heating profiles for advanced anaerobic digestion of municipal sludge. Water Res. 47, 5040–5051 (2013). 23866153 10.1016/j.watres.2013.05.055

[b20] Eskicioglu C., Terzian N., Kennedy K. J., Droste R. L. & Hamoda M. Athermal microwave effects for enhancing digestibility of waste activated sludge. Water Res. 41, 2457–2466 (2007). 17451781 10.1016/j.watres.2007.03.008

[b21] Mu Y., Zheng X.-J., Yu H.-Q. & Zhu R.-F. Biological hydrogen production by anaerobic sludge at various temperatures. Int. J. Hydrogen Energy 31, 780–785 (2006).

[b22] Wang Q., Jiang G., Ye L. & Yuan Z. Enhancing methane production from waste activated sludge using combined free nitrous acid and heat pre-treatment. Water Res. 63, 71–80 (2014). 24981745 10.1016/j.watres.2014.06.010

[b23] Carrère H. *et al.* Pretreatment methods to improve sludge anaerobic degradability: a review. J. Hazard. Mater. 183, 1–15 (2010). 20708333 10.1016/j.jhazmat.2010.06.129

[b24] Fu F., Dionysiou D. D. & Liu H. The use of zero-valent iron for groundwater remediation and wastewater treatment: A review. J. Hazard. Mater. 267, 194–205 (2014). 24457611 10.1016/j.jhazmat.2013.12.062

[b25] Mu Y., Yu H.-Q., Zheng J.-C., Zhang S.-J. & Sheng G.-P. Reductive degradation of nitrobenzene in aqueous solution by zero-valent iron. Chemosphere 54, 789–794 (2004). 14637335 10.1016/j.chemosphere.2003.10.023

[b26] Zhang J. *et al.* Bioaugmentation and functional partitioning in a zero valent iron-anaerobic reactor for sulfate-containing wastewater treatment. Chem. Eng. J. 174, 159–165 (2011).

[b27] Liu Y. *et al.* Effects of an electric field and zero valent iron on anaerobic treatment of azo dye wastewater and microbial community structures. Bioresour. Technol. 102, 2578–2584 (2011). 21167707 10.1016/j.biortech.2010.11.109

[b28] Zhang Y., Liu Y., Jing Y., Zhao Z. & Quan X. Steady performance of a zero valent iron packed anaerobic reactor for azo dye wastewater treatment under variable influent quality. Journal of Environmental Sciences 24, 720–727 (2012). 10.1016/s1001-0742(11)60803-6 22894108

[b29] Zhang Y., Jing Y., Quan X., Liu Y. & Onu P. A built-in zero valent iron anaerobic reactor to enhance treatment of azo dye wastewater. Water Sci. Technol. 63, 741–746 (2011). 21330722 10.2166/wst.2011.301

[b30] Liu Y. *et al.* Optimization of anaerobic acidogenesis by adding Fe0 powder to enhance anaerobic wastewater treatment. Chem. Eng. J. 192, 179–185 (2012).

[b31] Liu Y. *et al.* Enhanced azo dye wastewater treatment in a two-stage anaerobic system with Fe0 dosing. Bioresour. Technol. 121, 148–153 (2012). 22858479 10.1016/j.biortech.2012.06.115

[b32] Feng Y., Zhang Y., Quan X. & Chen S. Enhanced anaerobic digestion of waste activated sludge digestion by the addition of zero valent iron. Water Res. 52, 242–250 (2014). 24275106 10.1016/j.watres.2013.10.072

[b33] Zhang Y., Feng Y., Yu Q., Xu Z. & Quan X. Enhanced high-solids anaerobic digestion of waste activated sludge by the addition of scrap iron. Bioresour. Technol. 159, 297–304 (2014). 24657762 10.1016/j.biortech.2014.02.114

[b34] Lovley D. R. Organic matter mineralization with the reduction of ferric iron: a review. Geomicrobiol. J. 5, 375–399 (1987).

[b35] Ge H., Jensen P. D. & Batstone D. J. Increased temperature in the thermophilic stage in temperature phased anaerobic digestion (TPAD) improves degradability of waste activated sludge. J. Hazard. Mater. 187, 355–361 (2011). 21277081 10.1016/j.jhazmat.2011.01.032

[b36] Novak J., Verma N. & Muller C. The role of iron and aluminium in digestion and odor formation. Water Sci. Technol. 56, 59–65 (2007). 18025732 10.2166/wst.2007.705

[b37] Park C., Muller C. D., Abu-Orf M. M. & Novak J. T. The effect of wastewater cations on activated sludge characteristics: effects of aluminum and iron in floc. Water Environ. Res 78, 31–40 (2006). 16553164 10.2175/106143005x84495

[b38] Liu Y., Zhang Y., Quan X., Chen S. & Zhao H. Applying an electric field in a built-in zero valent iron – Anaerobic reactor for enhancement of sludge granulation. Water Res. 45, 1258–1266 (2011). 20965541 10.1016/j.watres.2010.10.002

[b39] Chen Y., Cheng J. J. & Creamer K. S. Inhibition of anaerobic digestion process: A review. Bioresour. Technol. 99, 4044–4064 (2008). 17399981 10.1016/j.biortech.2007.01.057

[b40] Park C. M. & Novak J. T. The effect of direct addition of iron (III) on anaerobic digestion efficiency and odor causing compounds. Water Sci. Technol. 68, 2391–2396 (2013). 24334887 10.2166/wst.2013.507

[b41] Gutierrez O., Park D., Sharma K. R. & Yuan Z. Iron salts dosage for sulfide control in sewers induces chemical phosphorus removal during wastewater treatment. Water Res. 44, 3467–3475 (2010). 20434190 10.1016/j.watres.2010.03.023

[b42] Ganigue R., Gutierrez O., Rootsey R. & Yuan Z. Chemical dosing for sulfide control in Australia: an industry survey. Water Res. 45, 6564–6574 (2011). 22018528 10.1016/j.watres.2011.09.054

[b43] Batstone D. J., Tait S. & Starrenburg D. Estimation of hydrolysis parameters in full-scale anerobic digesters. Biotechnol. Bioeng. 102, 1513–1520 (2009). 18988267 10.1002/bit.22163

[b44] Carballa M., Duran C. & Hospido A. Should we pretreat solid waste prior to anaerobic digestion? An assessment of its environmental cost. Environ. Sci. Technol. 45, 10306–10314 (2011). 22040018 10.1021/es201866u

[b45] Ge H., Jensen P. D. & Batstone D. J. Temperature phased anaerobic digestion increases apparent hydrolysis rate for waste activated sludge. Water Res. 45, 1597–1606 (2011). 21185054 10.1016/j.watres.2010.11.042

[b46] Donoso-Bravo A., Pérez-Elvira S. I. & Fdz-Polanco F. Application of simplified models for anaerobic biodegradability tests. Evaluation of pre-treatment processes. Chem. Eng. J. 160, 607–614 (2010).

[b47] Rao M. S., Singh S. P., Singh A. K. & Sodha M. S. Bioenergy conversion studies of the organic fraction of MSW: assessment of ultimate bioenergy production potential of municipal garbage. Applied Energy 66, 75–87 (2000).

