# Supplementary material for: Zero Valent Iron Significantly Enhances Methane Production from Waste Activated Sludge by Improving Biochemical Methane Potential Rather Than Hydrolysis Rate
Source: Sci Rep. 2015 Feb 5;5:8263. doi: 10.1038/srep08263 (PMC4317694; doi:10.1038/srep08263)
Supplement: Supplementary Information — SI for Zero Valent Iron Significantly Enhances Methane Production from Waste Activated Sludge by Improving Biochemical Methane Potential Rather Than Hydrolysis Rate [file srep08263-s1.docx]

# Supplementary materials

# Zero Valent Iron Significantly Enhances Methane Production from Waste Activated Sludge by Improving Biochemical Methane Potential Rather Than Hydrolysis Rate

# Yiwen Liu^a^, Qilin Wang^a^, Yaobin Zhang^b,^*, Bing-Jie Ni^a,^*

# ^a^Advanced Water Management Centre, The University of Queensland, St Lucia, QLD 4072, Australia

^b^Key Laboratory of Industrial Ecology and Environmental Engineering, Ministry of Education, School of Environmental Science and Technology, Dalian University of Technology, Dalian 116024, China

***Corresponding authors:**

Yaobin Zhang, P +86 411 84706460; F +86 411 84706263; E-mail: [zhangyb@dlut.edu.cn](mailto:zhiguo@awmc.uq.edu.au)

Bing-Jie Ni, P +61 7 33463230; F +61 7 33654726; E-mail: b.ni@uq.edu.au

**The following is included as additional supplementary materials for this paper:**

- Economic analysis of the ZVI-based technology for enhancing methane production
- Table S1: The estimated parameter values of k,_rapid_, k,_slow_, B_0,rapid_ and B_0,slow_ from Experiment I and Experiment II using two-substrate model
- Table S2. Economic analysis of the ZVI-based technology for enhancing methane production in WWTP
- Additional references in supplementary materials

**Economic analysis of the ZVI-based technology for enhancing methane production**

A desktop scaling-up study on a full-scale wastewater treatment plant (WWTP) with a 400,000 population equivalent (PE) and with an anaerobic digester at a hydraulic retention time (HRT) of 20 d was conducted to evaluate the potential economic benefit of the ZVI-based technology. A system with an annual methane production of approximately 294,700 kg CH_4_ was used as a control. A system with ZVI addition at 10 g/L was designed to obtain a 29% increase in methane production (i.e. 380,000 kg CH_4_ per annum). The methane produced was considered to be combusted in a cogeneration plant in order to produce both power and heat (power generation efficiency of 40% and heat generation efficiency of 50%).^1^ The costs/benefits caused by the introduction of ZVI addition were estimated, as summarized in Table S2.

**Table S1: The estimated parameter values of k,_rapid_, k,_slow_, B_0,rapid_ and B_0,slow_ from Experiment I and Experiment II using two-substrate model**

| Parameters  (unit) | k,_rapid_  (d^-1^) | B_0,rapid_  (L CH_4_/kg VS) | k,_slow_  (d^-1^) | B_0,slow_  (L CH_4_/kg VS) |
| --- | --- | --- | --- | --- |
| **Experiment I** |  |  |  |  |
| 0g/L Fe powder | 0.083 | 249 | 0 | 0 |
| 1g/L Fe powder | 0.083 | 271 | 0 | 0 |
| 4g/L Fe powder | 0.083 | 300 | 0 | 0 |
| **Experiment II** |  |  |  |  |
| 0g/L Fe scrap  10 g/L Fe powder | 0.073  0.072 | 214  240 | 0  0 | 0  0 |
| 10g/L clean Fe scrap | 0.072 | 262 | 0 | 0 |
| 10g/L rusty Fe scrap | 0.071 | 275 | 0 | 0 |

**Table S2. Economic analysis of the ZVI-based technology for enhancing methane production in WWTP**

| **General parameter** | | **Values** |
| --- | --- | --- |
| Size of the WWTP (Population equivalent - PE) | | 400,000 |
| Decay coefficient of the heterotrophic biomass (d^-1^) | | 0.2^a^ |
| Decay coefficient of the nitrifying biomass (d^-1^) | | 0.1^a^ |
| Yield coefficient of the heterotrophic biomass (g COD/g COD) | | 0.625^a^ |
| Yield coefficient of the nitrifying biomass (g COD/g N) | | 0.24^a^ |
| Fraction of inert COD generated in biomass decay (g COD/g COD) | | 0.2^a^ |
| Mixed liquor suspended solid concentration in the bioreactor (mg/L) | | 4,000 |
| Mixed liquor volatile suspended solid concentration in the bioreactor (mg/L) | | 3,200 |
| Sludge retention time (SRT) in the bioreactor of the WWTP (d) | | 15 |
| Solids content in thickened WAS | | 5% |
| Solids content in dewatered WAS | | 15% |
| HRT in the anaerobic digester (d) | | 20 |
| Methane calorific value (kwh/kgCH_4_) | | 16 |
| Power price ($/kwh) | | 0.12 |
| Conversion efficiency of methane to heat | | 50%^b^ |
| Conversion efficiency of methane to power | | 40%^b^ |
| Cost of WAS transport and disposal ($/wet tonne) | | 55 |
| Transport cost of rusty iron scrap ($/tonne) | | 15 |
| Period over which capital costs are annualised (*i.e.* Lifetime) (year) | | 20 |
| Interest applied for initial capital expenditure | | 8.5% |
| Energy generation associated CO_2_ emission (kgCO_2_/kwh) | | 1.05 |
| **Control system** | Methane production (kg CH_4_/y) | 294,700 |
|  | Volume of the anaerobic digester (m^3^) | 3,350 |
|  | WAS removal in the anaerobic digester (on a dry VS basis) | 34% |
| **System with rusty iron scrap addition** **(economic analysis)** | Methane production (kgCH_4_/y) | 380,000 |
|  | Volume of the anaerobic digester (m^3^) | 3,350 |
|  | WAS removal in the anaerobic digester (on a dry VS basis) | 44% |
|  | Annual extra heat production from methane conversion (compared to the control system) (kwh/y) | 693,000 |
|  | Annual extra power production from methane conversion (compared to the control system) (kwh/y) | 555,000 |
|  | Concentration of rusty iron scrap in the anaerobic digester (kg/m^3^) | 10 |
|  | Transportation of rusty iron scrap (times/y) | 3 |
|  | Cost of rusty iron scrap transportation ($/y) | 1,500 |
|  | Cost of Chamber ($/y) | 7,500 |
|  | **ZVI-associated cost ($/y)** | **9,000** |
|  | **Annual reduced WAS transport and disposal cost (compared to the control system) ($/y)** | **90,000** |
|  | **Annual extra obtained benefit (compared to the control system) due to the extra heat and power generation($/y)** | **150,000** |
|  | **Annual saving ($/y)** | **231,000** |

^a^ Reference (2); and ^b^ Reference (1)

**References**

(1) Carballa, M.; Duran, C.; Hospido, A. Should we pretreat solid waste prior to anaerobic digestion? an assessment of its environmental cost. *Environ. Sci. Technol.* **2011**, *45* (24), 10306-10314.

(2) Metcalf and Eddy. *Wastewater Engineering: Treatment and Reuse*. McGraw-Hill Inc.: **2003.**
